# Acute serum free thiols: a potentially modifiable biomarker of oxidative stress following traumatic brain injury

**DOI:** 10.1007/s00415-022-11240-6

**Published:** 2022-07-01

**Authors:** Koen Visser, Harm Jan van der Horn, Arno R. Bourgonje, Bram Jacobs, Martin H. de Borst, Pieter E. Vos, Marian L. C. Bulthuis, Harry van Goor, Joukje van der Naalt

**Affiliations:** 1 Department of Neurology, University of Groningen, University Medical Center Groningen, Hanzeplein 1, 9713 GZ Groningen, The Netherlands; 2 Department of Gastroenterology and Hepatology, University of Groninger, University Medical Center Groningen, Hanzeplein 1, 9713 GZ Groningen, The Netherlands; 3 Department of Internal Medicine, Division of Nephrology, University of Groningen, University Medical Center Groningen, Hanzeplein 1, 9713 GZ Groningen, The Netherlands; 4 Department of Neurology, Slingeland Hospital, 7009 BL Doetinchem, The Netherlands; 5 Department of Pathology and Medical Biology, University of Groningen, University Medical Center Groningen, Hanzeplein 1, 9713 GZ Groningen, The Netherlands

**Keywords:** Free radicals, Redox, Markers, Outcome, Head injury

## Abstract

Serum concentrations of free thiols (key components of the extracellular antioxidant machinery) reflect the overall redox status of the human body. The objective of this exploratory study was to determine the concentrations of serum free thiols in the acute phase after traumatic brain injury (TBI) and their association with long-term outcome. In this observational cohort study, patients with TBI of various severity were included from a biobank of prospectively enrolled TBI patients. Further eligibility criteria included an available blood sample and head computed tomography data, obtained within 24 h of injury, as well as a functional outcome assessment (Glasgow Outcome Scale Extended (GOSE)) at 6 months post-injury. Serum free thiol concentrations were markedly lower in patients with TBI (*n* = 77) compared to healthy controls (*n* = 55) (mean ± standard deviation; 210.3 ± 63.3 vs. 301.8 ± 23.9 μM, *P* < 0.001) indicating increased oxidative stress. Concentrations of serum free thiols were higher in patients with complete functional recovery (GOSE = 8) than in patients with incomplete recovery (GOSE < 8) (median [interquartile range]; 235.7 [205.1–271.9] vs. 205.2 [173–226.7] μM, *P* = 0.016), suggesting that patients with good recovery experience less oxidative stress in the acute phase after TBI or have better redox function. Acute TBI is accompanied by a markedly lower concentration of serum free thiols compared to healthy controls indicating that serum free thiols may be a novel biomarker of TBI. Future studies are warranted to validate our findings and explore the clinical applicability and prognostic capability of this candidate-biomarker.

## Introduction

Traumatic brain injury (TBI) is one of the most important causes of neurological morbidity worldwide, with an annual global incidence estimated at 50 million cases [1]. For severe TBI, early mortality can be as high as 40% [2]. Mild TBI (mTBI), which constitutes 80–90% of all TBIs, often presents with more subtle non-life threatening clinical alterations at the emergency department (ED) [3]. Nevertheless, approximately 40% of mTBI patients have not returned to their pre-injury functioning by 6 months after the initial head injury [4–6]. For TBI in general, and especially for mTBI, long-term outcome is still difficult to predict using acute injury parameters. Serum biomarkers are needed to bridge this gap.

A biomarker is defined as an indicator of the state of a biological process (e.g. concentrations of biochemicals measured in blood). A change in the concentration of a serum biomarker, therefore, provides a non-invasive insight into in vivo (patho)physiological responses following TBI. Many serum biomarkers have already been investigated and can be classified into markers of primary tissue injury (neuronal, axonal, or glial damage) or of secondary injury processes (e.g. neuroinflammation and oxidative stress) [7]. The clinical utility of primary injury markers has been thoroughly investigated in large-scale prospective cohort studies, whereas the value of secondary injury markers in TBI is largely unknown [8]. The limited knowledge on the clinical utility of markers of secondary injury is a consequence of small study populations with distinctly different inclusion criteria, methods of quantifying concentrations of the secondary injury markers, and pre-analytical methods [9].

Despite the significant amount of research data on primary injury biomarkers, translation to clinical use is limited, most likely owing to the complexity of the pathophysiology of TBI which is not captured by an individual marker [1]. Biomarker research in TBI has recently shifted towards biomarker panels to address this issue. A panel consisting of markers of primary and secondary processes may capture the specific injury profile of the individual patient allowing the panel to be generalizable to the TBI population, increasing clinical applicability [10]. Before this can be achieved, research is required on additional biomarkers of secondary injury.

Oxidative stress is a well-recognized secondary injury process [11]. This occurs when reactive species, which are damaging to lipids, DNA, and proteins, overwhelm endogenous antioxidants. Oxidative stress also acts in synergy with neuroinflammation, another key component of secondary injury after TBI [12]. Serum free thiols are one of the most important groups of antioxidants and reflect the systemic redox state [13]. A significant lower concentration of serum free thiols has been observed in a variety of oxidative stress-mediated human diseases such as non-alcoholic fatty liver disease (NAFLD), chronic heart failure (CHF), and inflammatory bowel disease (IBD) [14–16]. It is also known that free thiols are receptive to therapeutic modulation, providing a potential novel treatment pathway in patients with reduced concentrations of serum free thiols [16].

In this exploratory study, we investigate the utility of serum free thiols as a TBI biomarker. We hypothesized that concentrations of serum free thiols are reduced in patients with TBI compared to healthy controls. Furthermore, we proposed that TBI patients with higher concentrations of serum free thiols in the acute phase after TBI may show more favourable outcome, which might indicate free thiols to be a potential novel modifiable prognostic biomarker of TBI.

## Materials and methods

The reporting of the current study conforms to the EQUATOR guideline: the Strengthening of the Reporting of Observational Studies in Epidemiology (STROBE) [17].

### Subjects and study design

This study is a retrospective study of data obtained from the biobank of the prospective Radboud University Brain Injury Cohort (RUBIC) study. A complete description of the RUBIC study along with inclusion and exclusion criteria is described elsewhere [18].

In the current study, TBI patients of all severities, admitted to the ED of the Radboud University Medical Center (Radboudumc) from 1 January 2006 till 31 December 2009 were eligible for inclusion. Additional inclusion criteria were the availability of blood samples and head computed tomography (CT) data obtained within 24 h after trauma. Furthermore, functional outcome assessment using the Glasgow Coma Scale Extended (GOSE) had to be performed at 6 months post-trauma. Serum samples from healthy non-TBI controls were obtained from a subcohort of the TransplantLines biobank which contains pre-donation samples of living kidney donors [PSI-UMCG (IRB no. 2008/279)] [19]. Controls were algorithmically selected based on matching with the gender and age of TBI patients (a tolerance factor of ± three years was applied). Due to several younger patients in the TBI group, matching was suboptimal.

### Standards protocol approvals, registrations, and patient consents

Approval for the RUBIC study was obtained from the ethical committee of the Radboudumc (AMO 04/064 and CMO 2004/025) and for the PSI-UMCG biobank from the UMCG ethical committee (IRB no. 2008/279). Written informed consent was obtained from all participants of both biobanks (or guardians of participants). All procedures were carried out in accordance with the 2013 Helsinki Declaration.

### Data collection

The criteria for diagnosing TBI as mild, moderate, or severe were previously described [18]. Polytrauma was considered to exist in patients with an Abbreviated Injury Score (AIS) > 2 in two different body regions or an Injury Severity Score extracranial (ISSe) > 15 [20]. Isolated TBI was defined as TBI without the presence of polytrauma. TBI-specific clinical characteristics were also recorded, including the presence and duration of loss of consciousness (LOC) and/or post-traumatic amnesia (PTA), hypoxia (SaO_2_ < 90% or PaO_2_ <  = 8kpa), and hypotension (systolic blood pressure < 90 mmHg) at ED admission.

### Head CT

Head CT scans were obtained at ED admission and interpreted by two qualified neurologists according to the Trauma Coma Databank (TCDB) criteria [21]. To facilitate analysis, CT outcome was dichotomized into CT-negative (TCDB category 1) and CT-positive (TCDB categories 2–6).

### Functional outcome assessment

Functional outcome was assessed at 6 months according to the Glasgow Outcome Scale Extended (GOSE) at the out-patient clinic or during consultation by telephone [22]. When a patient was dismissed from follow-up before 6 months because of favourable GOSE score 7–8, this observation was carried forward and considered a definitive outcome. To increase power, GOSE scores were dichotomized into incomplete recovery (GOSE < 8) and complete recovery (GOSE = 8).

### Blood sampling and laboratory methods

Blood samples were taken at hospital admission and allowed to clot for a maximum of 30 min at room temperature. Samples were than processed for serum (centrifuged at 1000 × *g* for 10 min), and samples were moved to long-term storage according to local laboratory regulations. In 2014, serum samples were transported from the Radboudumc to the UMCG where they were stored at − 80 °C until analysis. Serum samples did not undergo any freeze–thaw cycles. The samples of healthy controls were collected and processed for serum (centrifuged at 1100 × *g* for 10 min) and stored at − 80 °C until analysis.

Concentrations of serum free thiols were measured blinded to group and in triplicate according to previously described methods with few modifications [23]. Upon thawing, samples were fourfold diluted using 0.1 M Tris buffer (pH 8.2). Background absorption was measured at 413 nm using the Varioskan microplate reader (Thermo Scientific, Breda, the Netherlands), along with a reference measurement at 630 nm. Subsequently, 20 μL 1.9 mM 5,5′-dithio-bis 2-nitrobenzoic acid (DTNB, Ellman’s reagent, CAS no. 69-78-3, Sigma-Aldrich Corporation, St. Louis, MO, USA) in 0.1 M phosphate buffer (pH 7.0) was added to samples. After an incubation time of 20 min at room temperature, absorbance was measured again. Final concentrations of serum free thiols were established by parallel measurement of an L-cysteine (CAS no. 51-90-4, Fluka Biochemika, Buchs, Switzerland) calibration curve (range from 15.6 to 1000 μM) in 0.1 M Tris/10 mM EDTA (pH 8.2). The coefficients of variation (intraplate and interplate) were all below 10%.

### Statistical methods

Assessment of normality was performed visually using histograms and normal probability plots (Q–Q plots). Group differences were assessed using independent sample *t* test and one-way ANOVA in case of normally distributed variables. Mann–Whitney *U* tests and Kruskal–Wallis tests were used in case of non-normally distributed continuous variables. Dunn’s or Tukey’s tests were used for post hoc analyses in case of significant ANOVAs or Kruskal–Wallis tests.

Correlations of demographic and clinical variables with serum free thiol concentrations were assessed using Pearson’s correlation coefficients for continuous data and Spearman correlations for categorical or nonparametric data. Furthermore, univariate binary logistic regression analysis was performed to identify the association between concentrations of serum free thiols and clinical variables (as independent variables) with functional outcome (as dependent variable). Odds ratios (OR) with 95% confidence intervals (CIs) were reported. Statistical analysis was performed using Python (v3.9.0) and associated libraries. Similarly, figures were created with the Python package matplotlib (v3.4.2) and then completed using Inkscape (v1.0.2). Overall, two-tailed *p* values ≤ 0.05 were considered statistically significant.

### Data availability

The data that support the findings of this study are available on reasonable request to the corresponding author.

## Results

### Study population

**Fig. 1:**
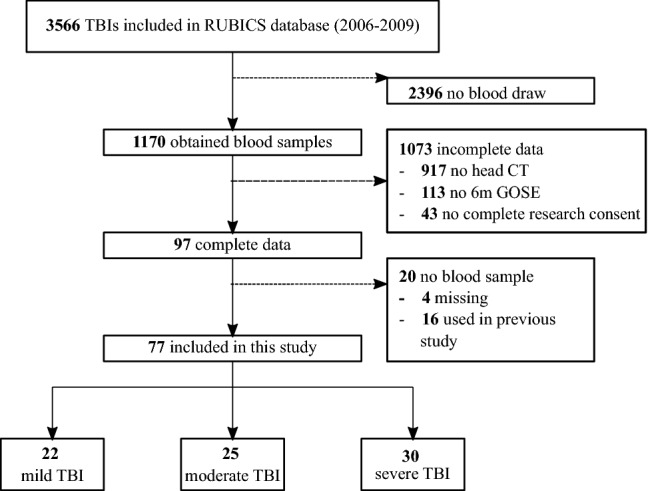
Flowchart of patient inclusion. *RUBICS* Radboud University Nijmegen Brain Injury Cohort Study, *TBI* traumatic brain injury, *CT* computed tomography, *GOSE* Glasgow Outcome Scale Extended

The final study cohort consisted of 77 TBI patients (Fig. 1). Demographics and clinical characteristics are listed in Table 1. Fifty-five (55) healthy controls (HC) were included. Healthy controls were significantly older than TBI patients (mean ± standard deviation; 42.7 ± 16.3 vs. 50.0 ± 12.0 years, *P* < 0.05). The proportion of female subjects in the TBI group were comparable to that of the HC group (38.9% vs. 38.2%). The median time to blood draw post-TBI was 77.5 min (max, 675 min).

**Table 1:** Demographic and clinical characteristics of 77 traumatic brain injury (TBI) patients and 55 healthy controls (HC)

	Mild TBI *n* = 22	Moderate TBI *n* = 25	Severe TBI *n* = 30	HC *n* = 55
Age^a^	47.7 (16.3)	44.7 (18.7)	37.9 (15.6)	50.0 (12.0)
Gender—female	8 (36.4%)	13 (52.0%)	9 (30.0%)	21 (38.2%)
Serum free thiols, μM^a^	225.9 (60.8)	209.7 (77.1)	199.2 (50.9)	301.8 (32.9)
Trauma mechanism				
Traffic	9 (40.9%)	16 (64.0%)	20 (66.7%)	–
Falls	10 (45.5%)	7 (28.0%)	5 (17.2%)	–
Sports	3 (13.6%)	1 (4.0%)	2 (6.7%)	–
Violence	–	–	2 (6.7%)	–
Other/unknown	–	1 (4.0%)	1 (3.3%)	–
GCS^b^	15 (13–15)	11 (9–12)	3 (3–8)	–
LOC	12 (54.6%)	19 (76.0%)	30 (100%)	–
PTA	14 (63.6%)	23 (92.0%)	29 (96.7%)	–
ISS^a^	13.8 (9.1)	18.8 (12.2)	36.5 (10.5)	–
ISSe^a^	9.6 (9.8)	8.3 (12.1)	18.9 (12.7)	–
AISH^b^	2 (1–4)	3 (2–4)	5 (4–5)	–
Polytrauma	7 (31.8%)	7 (28.0%)	22 (73.3%)	–
Hypotension	–	2 (8.0%)	3 (10.0%)	–
Hypoxia	1 (4.6)	3 (12.0%)	1 (3.3%)	–
Time to sample, min^b^	100 (10–675)	75 (28–600)	90 (10–470)	–
CT-positive	4 (18.2%)	16 (64.0%)	28 (93.3%)	–
Complete recovery (GOSE 8)	13 (59.1%)	10 (40.0%)	5 (16.7%)	–

*GCS* Glasgow Coma Score, *LOC* loss of consciousness, *PTA* post-traumatic amnesia, *ISS* Injury severity score, *ISSe* Injury severity score extracranial, *AISH* Abbreviated Injury Score Head, *TCDB* Traumatic Coma Databank, *CT* computed tomography, *GOSE* Glasgow Outcome Scale Extended, *μM* micromolar, *min* minutes

*At least one pupil unreactive

^a^Mean (sd); ^b^median (min–max); all other variables: *n* (%)

### Group comparisons of serum free thiol concentrations

**Fig. 2:**
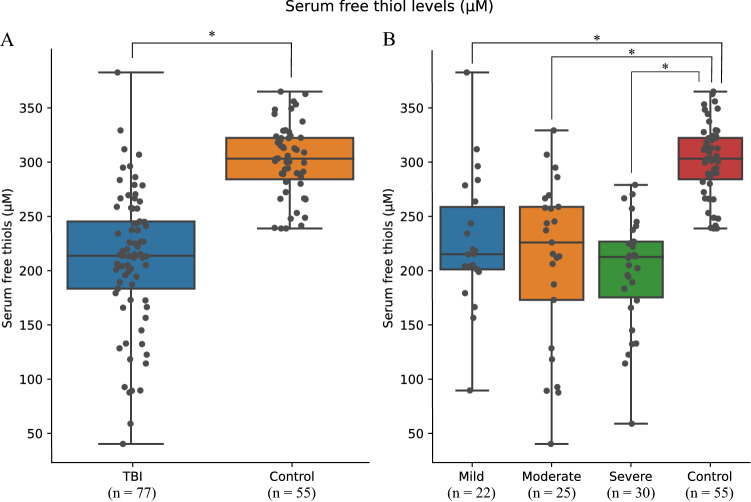
Comparing the concentration of serum free thiols between patients with TBI and healthy controls. **A** Serum free thiol levels are significantly decreased in traumatic brain injury compared with healthy controls; **B** levels of serum free thiols are also significantly lower in each TBI severity group compared to controls. *P < 0.001. *TBI* traumatic brain injury, *μM* micromolar

Serum free thiol concentrations were significantly decreased in the TBI group compared to healthy controls (mean ± SD; 210.3 ± 63.3 vs. 301.8 ± 23.9 μM, *P* < 0.0001). Post hoc analysis revealed that the concentrations of serum free thiols did not differ between TBI severity groups (Fig. 2).

### Associations between serum free thiols and clinical variables at emergency department admission

Serum free thiol concentrations were significantly decreased in patients with hypoxia, hypotension, or polytrauma (*P* < 0.001). Conversely, concentrations were higher in patients with complete recovery (GOSE = 8) at 6 months compared to those with incomplete recovery (median [interquartile range]; 235.7 [205.1–271.9] vs. 205.2 [173–226.7] μM, *P* = 0.016). (*r* = 0.28, *P* = 0.015). There were no correlations between serum free thiol concentrations and Glasgow Coma Scale (GCS) score (*r* = 0.15, *P* = 0.197), positive findings on CT (*r* = − 0.17, *P* = 0.153), and age (*r* = 0.17, *P* = 0.135) (Table 2).

**Table 2:** Median (interquartile range) of the initial serum concentrations of serum free thiols (μM) by clinical variables and outcomes

Variable	*n*	Serum free thiols	*P *value
TBI severity			0.385
Mild	22	215.1 (201.1–258.7)	
Moderate	25	226 (173–258.8)	
Severe	30	212 (175.3–226.7)	
Age			0.494
< 65	69	215.3 (183.4–257)	
≥ 65	8	209 (197.1–213.2	
Sex			0.193
Male	47	215 (192.6–258)	
Female	30	211.8 (166–244.6)	
CT			0.151
CT–	29	219.6 (201.8–266.6)	
CT +	48	212.6 (172.9–242.1)	
LOC			0.564
Yes	61	215.1 (201.6–239.9)	
No	16	213.7 (172.6–245.3)	
PTA			0.657
Yes	66	214.7 (184.3–254.2)	
No	11	206.3 (190.7–226.9)	
Polytrauma			0.001
Yes	36	202.1 (160.6–216.8)	
No	41	237.2 (206.3–269.5)	
Hypoxia*			0.001
Yes	8	123.3 (80.5–204.4)	
No	68	216.8 (193.2–257.4)	
Hypotension*			0.001
Yes	7	114.4 (74.1–147.8)	
No	69	215.4 (195.8–257.2)	
Incomplete recovery	49	205.2 (173–226.7)	–
Complete recovery	28	235.7 (205.1–271.9)	0.016
Overall	77	213.7 (183.4–245.3)	–

*P* values of Mann–Whitney *U* test in the case of two groups and Kruskal–Wallis in case of three groups

*μM* micromolar

*Includes suspected cases

**Fig. 3:**
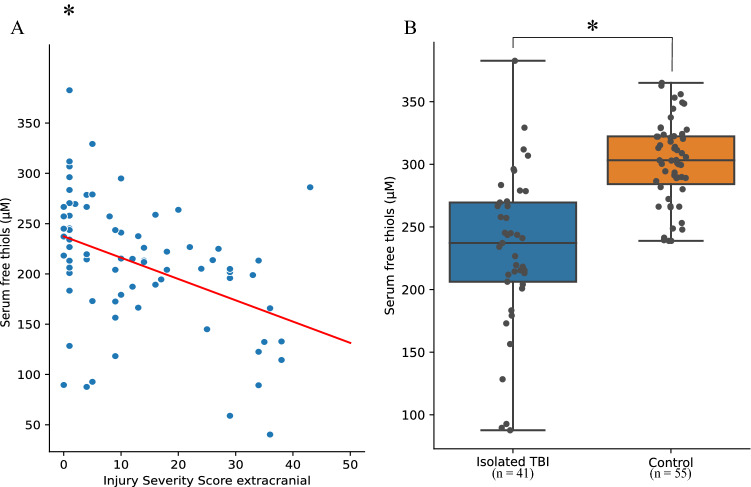
**A** Injury Severity Score extracranial (ISSe) correlates with serum free thiols (μM); **B** serum free thiols (μM) are significantly decreased in isolated TBI compared to healthy controls. *P = 0.01. *μM* micromolar, *TBI* traumatic brain injury

The presence of polytrauma correlated significantly with serum free thiol concentrations (*ρ* = 0.397, *P* = 0.001). Also, the ISSe was negatively correlated with concentrations of serum free thiols (*r* = − 0.42, *P* < 0.001). Concentrations of serum free thiols in isolated TBI were significantly lower compared to healthy controls (231.2 ± 62.6 vs. 301.8 ± 23.9 μM, *P* = 0.01) (Fig. 3). Patients with polytrauma had significantly reduced concentrations compared to isolated TBI (186.5 ± 56.0 vs. 231.2 ± 62.6 μM, *P* < 0.01). Levels of serum free thiols did not correlate with CT findings or TBI severity (*ρ* − 0.165 and − 0.147, respectively).

### Association of serum free thiols with outcome

Most patients had incomplete functional recovery (GOSE < 8) post-trauma (*n* = 49, 63.3%). The clinical characteristics that were significant determinants of complete recovery in univariate analysis included GCS, pupil reactivity, AIS head, ISS, ISSe, loss of consciousness, polytrauma, and a positive CT scan. The concentration of serum free thiols was also associated with complete recovery (Table 3).

**Table 3:** Unadjusted odds ratios of clinical and biochemical variables for complete recovery (GOS-E = 8) at 6 months after traumatic brain injury, using univariate logistic regression

Variable	OR (95% CI)
Age, year	1.01 (0.98–1.04)
Gender	2.12 (0.78–5.73)
GCS	1.18 (1.05–1.31)**
Hypotension*	0.66 (0.12–3.66)
Hypoxia*	0.54 (0.10–2.9)
AISH	0.49 (0.32–0.74)**
ISSe	0.95 (0.90–0.99)*
PTA	0.43 (0.12–1.55)
LOC	0.26 (0.08–0.81)*
CT	0.21 (0.08–0.57)**
Serum free thiols, μM	1.01 (1.0–1.02)*

*GOSE* Glasgow Outcome Scale Extended, *CI* confidence interval, *OR* odds ratio, *GCS* Glasgow Coma Score, *AISH* Abbreviated Injury Score Head, *ISSe* Injury Severity Score extracranial, *PTA* post-traumatic amnesia, *LOC* loss of consciousness, *CT* computed tomography, *μM* micromolar

*P < 0.05, **P < 0.001

## Discussion

This study demonstrates that serum free thiols are decreased in the acute phase following TBI compared to healthy controls, indicating that the acute phase of TBI is accompanied by oxidative stress. Furthermore, concentrations of serum free thiols were found to be higher in TBI patients with complete functional recovery at 6 months relative to those with incomplete recovery. These findings suggest that in patients with complete functional recovery less reactive species are generated in the acute phase post-trauma or alternatively that their antioxidant capacity is better. Free thiol concentrations negatively correlated with the ISSe indicating that levels are also influenced by oxidative stress due to extracranial injury. Nonetheless, free thiol concentrations remained significantly decreased in isolated TBI compared with HC, showing also a separate intracranial source of oxidative stress.

It is well established that oxidative stress is an important secondary injury mechanism of TBI [24]. In pre-clinical studies, evidence has been found for the presence of oxidative stress one-hour after head injury [25, 26]. Reactive oxygen species (ROS) and reactive nitrogen species (RNS) are the drivers of this response. Under physiological conditions, reactive species are involved in signalling cascades, allowing for complex control of protein structure and function [13]. Following TBI, however, glutamate released from the primary mechanical injury damages mitochondria releasing excessive reactive species. Next, a self-perpetuating cycle is formed as the excessive release of reactive species activates the neuroinflammatory response, consequently generating additional reactive species by the activated immune effector cells of the brain [12, 27]. When the quantity of released reactive species disbalances the homeostatic balance with endogenous antioxidants, secondary injury occurs owing to damaging effects of reactive species on lipids, proteins, and DNA, molecules found in most structures of the brain [28].

Due to the detrimental pathophysiological consequences of oxidative stress, studies have examined biomarkers to facilitate the understanding of the mechanisms of this response. However, few biomarkers of oxidative stress have thus far been identified for TBI or have clinical utility. Currently investigated biomarkers can generally be divided into two categories: by-products of oxidative reactions or reflecting the depletion of specific antioxidants. 4-Hydroxy-2-nonenal (4-HNE) a marker of lipid peroxidation and the nitrosative stress marker 3-nitrotyrosine (3-NT) are by-products commonly used in preclinical models [25, 28]. In the clinical settings, increased levels of 3-NT were found in the cerebrospinal fluid of 7 out of 10 TBI patients but only in two controls [29]. However, often these markers do not correlate with TBI severity or outcome, limiting clinical utility [28].

The findings of this current study show that serum free thiols are a potential candidate-biomarker of oxidative stress in clinical practice for TBI. Previously, it has been shown that serum free thiol concentrations were borderline different between controls and a population consisting of 24 largely mTBI patients [30]. In our study, however, there is a clear reduction in serum free thiols relative to controls across the full severity spectrum of TBI. A potential reason for this finding of a greater reduction in levels of serum free thiols might be that we included patients with major polytrauma and many patients with severe TBI. Interestingly, there was also a significant difference in the concentration of free thiols in the healthy controls and isolated TBI populations. A probable reason for the finding that serum free thiols outperform other markers of oxidative stress might be that it reflects whole-body redox status as a major component of the reactive species interactome, whereas previously investigated markers reflect spill-over products originating from individual upstream redox processes [13]. It would be interesting to see how serum free thiols compare to brain specific markers such as glial fibrillary acidic protein (GFAP), S100 calcium binding protein B (S100B), and Ubiquitin carboxy-terminal hydrolase L1 (UCH-L1), arguably the most promising biomarkers to date [31, 32]. This comparison would be especially interesting in a population consisting of only mTBI patients as free radical generation is an early occurring process which does not rely on measurable levels of cellular damage (often not present after mTBI).

The significant correlation between concentrations of serum free thiols and extracranial injury (ISSe) demonstrates that serum free thiols, like most currently investigated TBI biomarkers, are not brain specific [33]. Therefore, the contribution of intracranial sources to biomarker concentrations measured in fluid compartments more distant from the brain (e.g. blood, urine, or saliva vs. cerebrospinal fluid or micro dialysate fluid) is difficult to establish. Despite this drawback, our results indicate that serum free thiols are not solely a general marker of extracranial injury severity: (1) the concentration of free thiols in the mild TBI group is higher than in the moderate TBI group even though the average ISSe is higher in the mild TBI group, (2) patients with isolated head injury still have a significantly lower concentration of free thiols compared to health controls, indicating that part of the decrease in serum free thiols is due to free radicals produced by pathophysiological process within the brain, and (3) the ISSe of the severe TBI group is almost double that of the mild TBI group, yet there is no significant difference in free thiol concentrations. If free thiol concentrations were a true reflection of injury severity, a much larger concentration difference between the two groups could be expected.

The significant contribution of non-TBI trauma to the decline in serum free thiol concentrations of the TBI group is unsurprising. Various studies have demonstrated an important role of oxidative stress in trauma such as in multiple blunt trauma and abdominal trauma [34, 35]. A mechanism by which oxidative stress occurs after non-TBI trauma is through activation of neutrophils which can generate free radicals in a process called the ‘oxidative burst’ [36]. Despite the known relevance of oxidative stress to the pathophysiological mechanisms of many forms of non-TBI trauma, few studies have investigated serum free thiols in this context. One study quantified thiol concentration in paediatric trauma patients reporting a significant reduction in free thiol levels between the trauma group and healthy controls [37]. A similar reduction of thiol levels was found in patients with gunshot injury [38]. Although these studies are not comparable with the current study in terms of patient populations, sampling times and methods of quantifying free thiols they do reinforce the strong effect of non-TBI trauma on free thiol concentrations. Further studies should consider the use of non-TBI trauma controls to gather better insight into the contribution of intracranial sources of reactive species to the reduction in serum free thiol concentration.

This study is the first to assess the association of serum free thiols with long-term outcome after TBI. Our results might suggest that the concentrations of serum free thiols at the acute stage of TBI are reflective of long-term functional outcome as measured by the GOSE. A statistically significant higher concentration of free thiols was found in patients with complete recovery (GOSE = 8) compared to those with incomplete recovery (GOSE < 8).

Beyond diagnostic or prognostic utility, quantification of biomarkers can allow for disease characterization and identification of patients at risk for unfavourable long-term outcome who might benefit from targeted treatment [7]. For instance, free thiols might be used for identifying patients who could benefit from antioxidant therapy [16]. Furthermore, free thiols themselves, as strong antioxidants, may be a target of antioxidant therapies. N-acetylcysteine (NAC) is a FDA-approved antioxidant which has demonstrated capacity to increase free thiol concentrations [39, 40]. After further validation of serum free thiols as a biomarker, it would be interesting to assess the efficacy of NAC in patients with decreased serum free thiol concentrations in the acute phase post-trauma.

Although our results are promising, future prospective multicentre longitudinal cohort studies are required to evaluate the true clinical utility of serum free thiols as a biomarker of TBI. Longitudinal measures, preferably at acute, subacute, and chronic time points, will give a better indication of the temporal profile of oxidative stress following TBI and identify potential ‘therapeutic windows’ for antioxidant therapy. Knowledge on the temporal profile of serum free thiol concentrations after TBI is important as literature suggests a peak reduction of total antioxidant reserves five-seven days post-TBI [41]. The TBI samples of this study, obtained within 24 h of trauma, might therefore not fully reflect the entire scope of intracranial oxidative stress. Finally, it would be interesting to see how serum free thiols are correlated with other biomarkers of brain injury. Assessing this correlation may lead to the formation of biomarker panels, enabling the simultaneous assessment of the extent of both the primary and secondary injury processes that occur in the acute phase after TBI.

### Strengths and limitations

A major strength of this study is the well-optimized method of measuring serum free thiol concentrations. This technique, established in 1959, now provides reliable results of free thiol concentrations at the micromolar range [16, 23, 42]. Considering all coefficients of variation (intra and inter) in this study were below 10% exemplifies the reliability of this method. A further strength pertains to the well-characterized cohort of this study, with both early admission data but also available CT scans and outcome for all patients compromising the full spectrum of severity of TBI.

Most of the limitations of this study arise from the fact that the analysed TBI samples were collected from a pre-existent biobank. The collection and preparation of serum samples occurred many years ago [18]. We therefore had no influence on the preanalytical methods, for example, the processing for serum and storage conditions. Although preanalytical methods are protocolized in general, they may affect the stability of stored markers and thus the reliability of measured concentrations [43]. Despite using older samples, significant changes in the levels of free thiols in the samples of patients compared to healthy controls were found suggesting that the concentrations of serum free thiol remain stable even after long periods of storage. Interestingly, a recent study investigating the role of serum free thiols in renal transplant receivers reported a significant inverse association between sample storage time and serum free thiol concentrations [44]. The measured serum free thiol concentrations in the older TBI samples might be higher than if they were quantified immediately post-trauma as an effect of storage time. Following this reasoning, the findings of this study could be an underestimation of the true group difference of serum free thiols between TBI patients and healthy controls. Studies should verify this finding in samples that have not been stored for extended periods of time. For transparency, the preanalytical methods used are reported in the methods section.

A further limitation is a direct consequence of the eligibility criteria of this study. Patients were included if they had provided consent to use their blood samples for research purposes and additionally when data on head CT and long-term outcome data were available. This is a potential source of selection bias as patients with minor head injury or early mortality would most likely not have undergone a CT scan. As patient selection was based on data availability, there are some notable differences in demographic/clinical factors between the TBI severity groups: the extent of extracranial injury, age, gender, number of CT abnormalities, and outcome. Difference in factors such as extracranial injury, amount of CT abnormalities and distribution of outcomes is inherent to increased severity of TBI. The minor differences in age and gender may affect the concentrations of serum free thiols, decreasing the reliability of between TBI severity group comparisons of the concentrations. There is also a significant difference in ages between the TBI and HC populations with the HC being significantly older. Studies have demonstrated a decreased free thiol pool in older patients [45]. This means that the true group difference between HC and TBI patients is potentially underestimated.

Ultimately, this means that our findings of a single-centre study cannot be generalized to the entire TBI population. Future studies are needed to confirm our findings by measuring the concentration of serum free thiols in a large prospective cohort of consecutively enrolled patients with various severity of TBI. Furthermore, as future studies will likely have larger sample sizes, statistical techniques such as multivariate regression will be possible to demonstrate the true usefulness of serum free thiols as a biomarker of TBI outcome prediction.
